# Bone Loss at the Hip and Subsequent Mortality in Older Men: The Osteoporotic Fractures in Men (MrOS) Study

**DOI:** 10.1002/jbm4.10006

**Published:** 2017-07-10

**Authors:** Peggy M Cawthon, Sheena Patel, Susan K Ewing, Li‐Yung Lui, Jane A Cauley, Jennifer G Lyons, Lisa Fredman, Deborah M Kado, Andrew R Hoffman, Nancy E Lane, Kristine E Ensrud, Steven R Cummings, Eric S Orwoll, for the Osteoporotic Fractures in Men (MrOS) Study Research Group

**Affiliations:** ^1^ California Pacific Medical Center Research Institute San Francisco CA USA; ^2^ Department of Epidemiology and Biostatistics University of California San Francisco, San Francisco CA USA; ^3^ Department of Epidemiology Graduate School of Public Health University of Pittsburgh Pittsburgh PA USA; ^4^ Department of Epidemiology Boston University School of Public Health Boston MA USA; ^5^ Departments of Family Medicine & Public Health and Internal Medicine University of California San Diego CA USA; ^6^ Department of Medicine Stanford University Stanford CA; ^7^ Center for Musculoskeletal Health and Department of Internal Medicine University of California at Davis Sacramento CA USA; ^8^ Center for Chronic Disease Outcomes Research Minneapolis VA Health Care System Minneapolis MN USA; ^9^ Division of Epidemiology and Community Health University of Minnesota Minneapolis MN USA; ^10^ Department of Medicine University of Minnesota Minneapolis MN USA; ^11^ Oregon Health and Science University Portland OR USA

**Keywords:** DXA, ANALYSIS/QUANTITATION OF BONE, AGING, EPIDEMIOLOGY

## Abstract

Low bone mineral density (BMD) is associated with increased mortality risk, yet the impact of BMD loss on mortality is relatively unknown. We hypothesized that greater BMD loss is associated with increased mortality risk in older men. Change in femoral neck BMD was assessed in 4400 Osteoporotic Fractures in Men (MrOS) study participants with two to three repeat dual‐energy X‐ray absorptiometry scans over an average of 4.6 ± 0.4 (mean ± SD) years. Change in femoral neck BMD was estimated using mixed effects models; men were grouped into three categories of BMD change: maintenance (*n* = 1087; change ≥ 0 g/cm^2^); expected loss (*n* = 2768; change between 0 g/cm^2^ and <1 SD below mean change [>–0.034 g/cm^2^]); and accelerated loss (*n* = 545; change 1 SD below mean change or worse [≤–0.034 g/cm^2^]). Multivariate proportional hazards models adjusted for potential confounders estimated the risk of all‐cause mortality over 8.1 ± 2.8 years following visit 2. Mortality was centrally adjudicated by physician review of death certificates. At visit 1, mean age was 72.9 ± 5.5 years. Men who maintained BMD were less likely to die during the subsequent follow‐up period (33.7%) than men who had accelerated BMD loss (60.6%) (*p* < 0.001). Compared to men who had maintained BMD, those who had accelerated BMD loss had a 44% greater risk of mortality in multivariate‐adjusted models (HR, 1.44; 95% CI, 1.23 to 1.68). Compared to men who had maintained BMD, there was no significant difference in mortality risk for men with expected loss of BMD (36.9% died) (multivariate HR, 1.00; 95% CI, 0.89 to 1.13). Further adjustment for visit 1 or visit 2 BMD measurement did not substantially alter these associations. Results for total hip BMD were similar. In conclusion, accelerated loss of BMD at the hip is a risk factor for mortality in men that is not explained by comorbidity burden, concurrent change in weight, or physical activity. © 2017 The Authors. *JBMR Plus* is published by Wiley Periodicals, Inc. on behalf of the American Society for Bone and Mineral Research.

## Introduction

Low bone mineral density (BMD) is associated with mortality in older adults.^1^, ^2^, ^3^, ^4^ Both low BMD and bone loss are associated with fractures, which in turn are associated with mortality.^5^, ^6^, ^7^, ^8^, ^9^, ^10^ Thus, bone loss may be a marker for overall declining health and related to increased mortality. However, few studies have investigated the relation between bone loss and mortality, especially in men.^11^, ^12^ Previous reports also used simple linear change in BMD which may not have accounted for intraindividual and interindividual variability in BMD change, and these reports and did not account for BMD at both the start and the end of the change period as covariates. We have previously characterized hip bone loss using mixed effects linear regression in the Osteoporotic Fractures in Men (MrOS) cohort,^13^ and found that greater bone loss was a significant risk factor for fracture.^14^ Herein we tested the hypothesis that bone loss at the hip is independently associated with increased risk of mortality in older men, using data from MrOS.

## Materials and Methods

### The MrOS study

Between March 2000 and April 2002, the MrOS study enrolled 5994 men at six clinical sites across the United States as described.^15^ Men were eligible if they were aged 65 years or older, did not have bilateral hip replacements, and were able to walk without assistance. For these analyses, we used data from visit 1 and two follow‐up visits: the “sleep visit” (December 2003 to March 2005), an ancillary study to investigate sleep in a subcohort of participants; and “visit 2” (March 2005 to May 2006), a follow‐up visit for all surviving baseline participants.

### Assessment of BMD

At all study visits, participants who attended the clinic visit had hip dual‐energy X‐ray absorptiometry (DXA) scans completed on Hologic 4500 scanners (Hologic, Waltham, MA, USA) following centralized quality control procedures as described, including longitudinal review of phantom data and corrections for shift and/or drift in BMD values if needed.^13^ The coefficient of variation of the MrOS DXA scanners estimated using a central phantom ranged from 0.3% to 0.7% for the total hip (data not shown). The participant's right hip was scanned unless there was a fracture, implant, hardware, or other problem preventing the right hip from being scanned; in those instances, the left hip was scanned.

### Mortality ascertainment

Study participants were contacted via mailed questionnaire every 4 months after visit 1. Clinic staff was usually notified of a participant's death when following up on missed contacts. Deaths were centrally adjudicated by physician review of death certificates and hospital discharge summaries (when available) to broadly assign cause of death using the International Classification of Diseases, Ninth Revision codes as cardiovascular (CVD; 396.9–442, 996.71, 785.51), cancer (141.9–208.0), and other (non‐cancer non‐CVD). We included deaths through January 2016 in these analyses.

### Other measures

At all visits, the following variables were collected using identical methods. Weight was measured on balance beam or digital scales, and height was measured using wall‐mounted stadiometers. Participants self‐reported a physician diagnosis of medical conditions (see footnote, Table 1). Activity level was determined by the Physical Activity Scale for the Elderly (PASE).^16^ Race, smoking status, and self‐rated health status (excellent/good versus fair/poor/very poor) were by self‐report. Participants completed a battery of physical performance tests including ability and time to complete five repeated chair stands and walking speed (m/s) over 6 m.

**Table 1:** Characteristics of Participants at Visit 1 by Follow‐Up Mortality Status

	Survived (*n* = 2683)	Died (*n* = 1717)	*p*
Visit 1 femoral neck BMD (g/cm^2^)	0.795 ± 0.125	0.776 ± 0.126	<0.001
Visit 2 femoral neck BMD (g/cm^2^)	0.787 ± 0.127	0.756 ± 0.131	<0.001
Visit 1 total hip BMD (g/cm^2^)	0.972 ± 0.134	0.949 ± 0.14	<0.001
Visit 2 total hip BMD (g/cm^2^)	0.962 ± 0.137	0.924 ± 0.147	<0.001
Change in femoral neck BMD, visit 1 to visit 2 (%)	–1.29 ± 2.44	–2.32 ± 3.21	<0.001
Change in total hip BMD, visit 1 to visit 2 (%)	–1.23 ± 2.31	–2.56 ± 3.35	<0.001
Weight at visit 1 (kg)	83.7 ± 12.5	83.3 ± 13.9	0.297
Change in weight, visit 1 to visit 2 (%/year)	–0.3 ± 0.5	–0.5 ± 0.6	<0.001
BMI at visit 1 (kg/m^2^)	27.4 ± 3.6	27.5 ± 4.1	0.410
Excellent, good self‐reported health at visit 1	2465 (91.9)	1446 (84.2)	<0.001
At least one medical condition^a^	1963 (73.2)	1413 (82.3)	<0.001
Total PASE score at baseline	157.4 ± 67.0	142.8 ± 67.3	<0.001
Change in PASE, visit 1 to visit 2 (%/year)	–2.38 ± 3.62	–3.01 ± 6.93	<0.001
Age at visit 1 (years)	71.2 ± 4.6	75.5 ± 5.7	<0.001
White race	2384 (88.9)	1594 (92.8)	<0.001
History of any fracture before visit 1	1487 (55.4)	969 (56.4)	0.518
Alcoholic drinks per week at visit 1			<0.001
None (<12 drinks/year)	839 (31.31)	622 (36.27)	
Light (1–13 drinks/week)	1528 (57.01)	889 (51.84)	
Moderate/heavy (14+ drinks/week)	313 (11.68)	204 (11.9)	
Walking speed at visit 1 (m/s)	1.27 ± 0.2	1.17 ± 0.2	<0.001
Current smoker (versus former/never) at visit 1	76 (2.8)	57 (3.3)	<0.001
Chair stands performance at visit 1			<0.001
Quartile 1 (fastest)	766 (28.67)	310 (18.12)	
Quartile 2	688 (25.75)	386 (22.56)	
Quartile 3	658 (24.63)	426 (24.9)	
Quartile 4 (slowest)	535 (20.02)	548 (32.03)	
Unable	25 (0.94)	41 (2.4)	

Values are *n* (%) or mean ± SD as shown.

^a^ Medical conditions include: diabetes, stroke, hyperthyroidism, hypothyroidism, Parkinson's disease, heart attack, congestive heart failure, chronic obstructive pulmonary disease, cancer, hypertension, arthritis (osteoarthritis or rheumatoid), and angina pectoris.

### Analytic sample

The analytic sample included men who provided hip BMD measurements from both visit 1 and visit 2. Of the 5994 men enrolled in MrOS, 4400 men (73.4%) had DXA data for at least visit 1 and visit 2. Average time between visit 1 and visit 2 was 4.6 ± 0.4 years. Men who were excluded from the analyses due to missing BMD measures at follow‐up visits were older at visit 1, were less likely to report excellent/good health status, had lower activity levels by PASE, slower walking speed, weaker grip strength, and had lower BMD at the total hip and femoral neck than men who were included in the analyses (*p* < 0.001, data not shown).

### Statistical analysis

We categorized rate of change of BMD as reported.^13^, ^14^ We determined the association between rate of change in BMD and subsequent mortality.

#### Stage 1: Mixed effects models for determination of BMD change and categories of change (summary of previous reports)^13^, ^14^

Change in femoral neck and total hip BMD were determined for all participants with at least visit 1 and visit 2 repeat DXA assessments using random effects regression models (PROC MIXED procedure in SAS Version 9.2; SAS Institute, Cary, NC, USA).^(13)^ We included either two measures (visit 1 and visit 2) or three measures (visit 1, sleep, and visit 2) of BMD per participant, which allowed for more precise estimates of change. Random effects models account for between‐subject and within‐subject correlation between repeat measurements, and allow for each participant to have a unique estimated intercept (baseline BMD level) and estimated trajectory (change in BMD); age, and a quadratic term for age were included as the time variables. We grouped participants into three categories based on BMD change (that is, the estimated trajectory from the mixed effect model), for the femoral neck and total hip separately: maintained BMD (estimated change ≥ 0 g/cm^2^); expected loss (estimated change between 0 and 1 SD below mean change, −0.034 g/cm^2^ for femoral neck and −0.042 g/cm^2^ for total hip); and accelerated loss (estimated change ≥ 1 SD below mean change). We also analyzed change in BMD as a continuous variable.

#### Stage 2: Association between BMD change and subsequent risk of mortality

**Figure 1:**
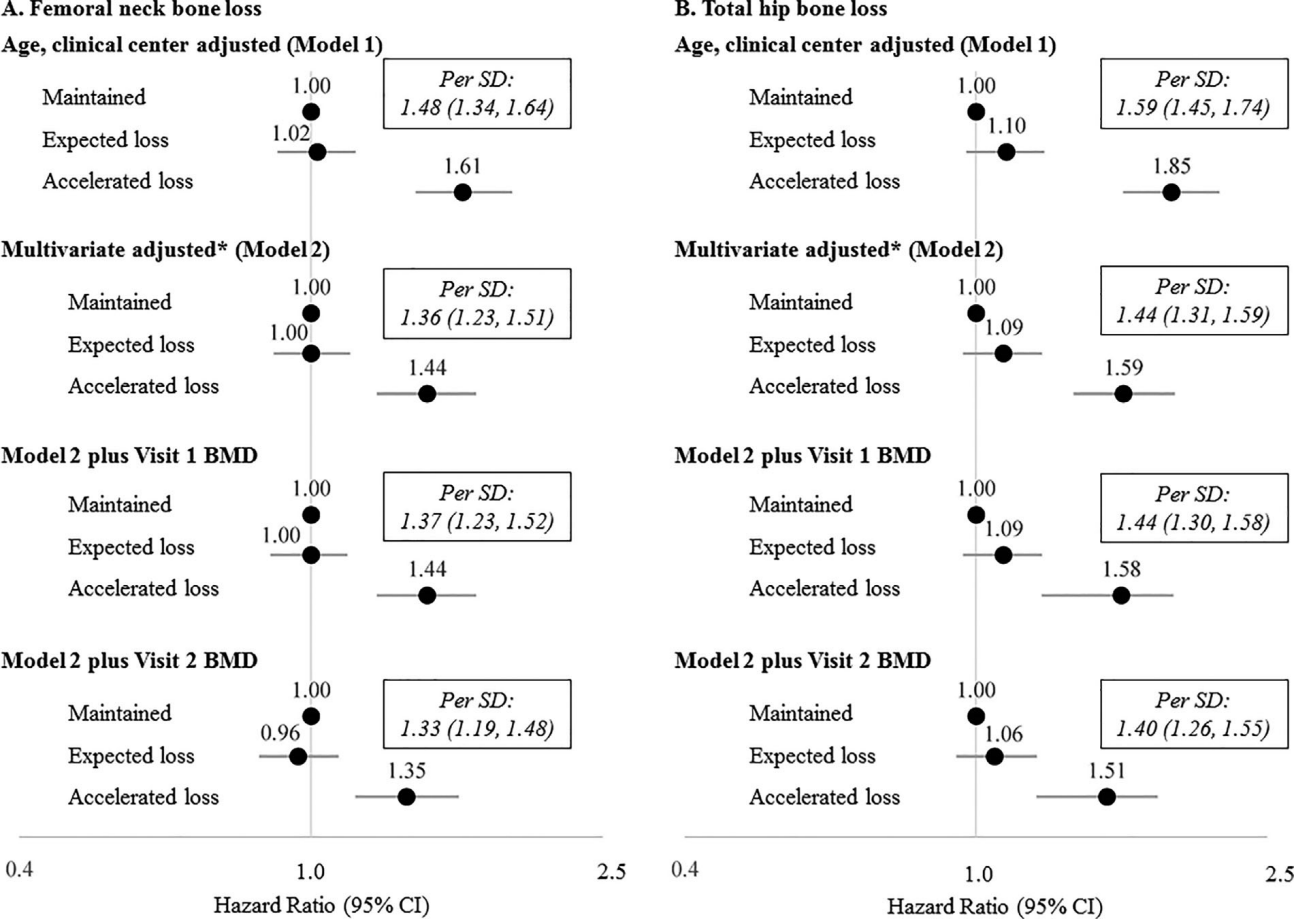
Change in bone mineral density at the femoral neck (*A*) or total hip (*B*) and hazard ratio for all‐cause mortality in older men over 8.1 years. *Models adjusted for adjusted for visit 1 age, clinic site, weight, physical activity, self‐reported heath, presence of at least one comorbid condition, smoking status, chair stands performance, concurrent change in weight, and concurrent change in self‐reported physical activity. A total of 1087 maintained femoral neck BMD of whom 366 died (33.7%); 2768 had expected loss of femoral neck BMD of whom 1021 died (36.9%); 545 had accelerated loss of femoral neck BMD of whom 330 died (60.6%), *p* < 0.001. A total of 1150 maintained total hip BMD; of whom 346 died (30.1%); 2648 had expected loss of total hip BMD of whom 984 died (37.2%); 602 had accelerated loss of total hip BMD of whom 387 died (64.3%), *p* < 0.001.

Relative risk of mortality after visit 2 was estimated as hazard ratios using Cox proportional hazards models, separately for femoral neck and total hip BMD. For each of the BMD sites, four sets of adjusted models were run: model 1: age‐ and clinical center adjusted; model 2: multivariate model (“model 2”, see adjustments, Fig. 1 footnote); model 3: multivariate model that further adjusted Model 2 for BMD at visit 1; model 4: multivariate model that further adjusted model 2 for BMD at visit 2.

## Results

Over 8.1 ± 2.8 years of follow‐up after visit 2, 1717 (39.0%) of the men died, including 574 (33.4% of the deaths) due to CVD death, 425 (24.8% of the deaths) due to cancer death, and 718 (41.8% of the deaths) due to other (non‐CVD, non‐cancer) death. Of those who maintained femoral neck BMD between visit 1 and visit 2 (*n* = 1087), 366 subsequently died (33.7%); of those with expected loss of femoral neck BMD (*n* = 2768), 1021 died (36.9%), and of those who had accelerated loss of femoral neck BMD (*n* = 545), 330 died (60.6%, *p* < 0.001) (Fig. 1 *A*). A similar pattern was observed for total hip BMD (see footnote, Fig. 1 *B*). Men who died were older at visit 1, had lower activity levels, greater comorbidity burden, walked slower, had worse chair stands performance, were more likely to be a current smoker and less likely to consume alcohol than those who survived (*p* < 0.001 for all) (Table 1). Compared to men who survived, those who died also had greater decline in activity level and greater weight loss between visit 1 and visit 2 (*p* < 0.001). There were no differences in visit 1 weight, BMI, or history of fracture before visit 1 by mortality status (*p* > 0.05). Characteristics of participants by category of femoral neck^(13)^ or total hip BMD^14^ change have previously been reported in MrOS.

In age‐ and clinical center‐adjusted models, men who had accelerated loss of femoral neck BMD had a 61% increased risk of mortality (HR, 1.61; 95% CI, 1.39 to 1.88, Fig. 1) compared to those who maintained femoral neck BMD, whereas men with expected loss of femoral neck BMD had a similar risk of mortality as those who maintained femoral neck BMD (HR, 1.02; 95% CI, 0.90 to 1.15). When bone loss was analyzed as a continuous variable, each SD additional loss of femoral neck BMD was associated with an increased risk of death (HR per SD decrease, 1.48; 95% CI, 1.34 to 1.64). In the multivariate model (model 2), adjustment for potentially confounding factors attenuated the association somewhat, such that men with accelerated loss of femoral neck BMD had a 44% greater risk of mortality than men who maintained femoral neck BMD (HR, 1.44; 95% CI, 1.23 to 1.68). Consistent with the age‐ and clinical center–adjusted models, in the multivariate models, no difference in mortality risk between men with expected loss of femoral neck BMD and men with maintenance of BMD was observed (HR, 1.00; 95% CI, 0.89 to 1.13). Further adjustment for visit 1 or visit 2 BMD did not substantially change the associations. Results for total hip BMD were similar. Results for cardiovascular mortality and “other” mortality followed similar patterns, although associations between BMD loss and cardiovascular mortality appeared somewhat stronger (Supporting Tables  1 and 2). There was no association between BMD loss and cancer mortality.

## Discussion

Men with accelerated bone loss at the femoral neck or total hip had a higher risk of mortality than men who maintained BMD. This association was not fully explained by confounding factors including comorbidity burden, change in activity or change in weight, nor was it explained by initial or final BMD level. However, men who had expected BMD loss did not have a greater risk of mortality than men who maintained BMD.

These results expand on previous findings in the MrOS study and others, and our results are consistent with previous reports that low BMD is associated with increased mortality.^1^, ^2^, ^3^, ^4^, ^17^, ^18^ However, they differ from a previous report that found no association between bone loss and mortality in men.^11^ This earlier study had relatively few events, and used a somewhat broad, dichotomous assessment of bone loss that was defined as 1% or greater/year. In our data, the increased risk of mortality was limited to men with the greatest bone loss. Thus, different approaches to quantify bone loss may have led to the discrepant findings between these reports.

Our study has many strengths. MrOS is a large, well‐characterized cohort of community dwelling older men with rigorous assessment of BMD and complete assessment of mortality. However, a few limitations must be noted. First, the cohort is mostly healthy men with relatively low minority participation, which may limit generalizability. Second, despite the comprehensive assessment of covariates in MrOS, some potentially confounding factors were either not measured (eg, some comorbidities were not assessed at visit 1, such as depression) or were not measured optimally (eg, self‐reported physical activity instead of objective measures). Thus, we cannot rule out uncontrolled confounding as a potential explanation for our results. Third, we have not estimated the attributable risk for mortality for each factor in our model; a more complete model assessing many competing risk factors for mortality would be more appropriate for such calculations and could be the subject of a separate report from this cohort. Finally, we have not formally tested whether the association between change in BMD and mortality is linear or if a threshold for mortality risk exists.

Change in BMD is not likely to cause death, but is a marker of an underlying biological and medical processes that increase mortality, and we propose that BMD loss may serve as a biomarker for the overall rate of aging. Evidence in genetically homogenous mice suggests that greater cortical bone thickness and preservation of endosteal perimeter is associated with longer murine lifespan.^19^ When our results are considered with the data from mice, this combined evidence does suggest that bone loss (or other traits related to bone health) may help identify those with a greater rate of aging, and higher mortality rates.

In conclusion, accelerated loss of BMD is a risk factor for mortality in MrOS. Future research should focus on identifying biological factors that lead to both rapid bone loss and increased risk of non‐cancer mortality.

## Disclosures

All authors state that they have no conflicts of interest.

## Supporting information

Supporting Table S1.

Supporting Table S1.
