# Supplementary material for: Bone Loss at the Hip and Subsequent Mortality in Older Men: The Osteoporotic Fractures in Men (MrOS) Study
Source: JBMR Plus. 2017 Jul 10;1(1):31–5. doi: 10.1002/jbm4.10006 (PMC5673261; doi:10.1002/jbm4.10006)
Supplement: Supplementary file 1 — Supporting Table S1. [file JBM4-1-31-s001.docx]

| Supplemental Table 1. Risk of cause-specific mortality by change in femoral neck BMD in older men. | | | | | | | | | | | | |
| --- | --- | --- | --- | --- | --- | --- | --- | --- | --- | --- | --- | --- |
|  | CVD Death | | | | Cancer Death | | | | Other Death (non cancer non CVD) | | | |
|  | BMD change category | | | per SD | BMD change category | | | per SD | BMD change category | | | per SD |
|  | Maintained | Expected | Accel. |  | Maintained | Expected | Accel. |  | Maintained | Expected | Accel. |  |
| Total n | 1087 | 2768 | 545 | 4400 | 1087 | 2768 | 545 | 4400 | 1087 | 2768 | 545 | 4400 |
| N (%) died | 118  (10.9) | 334 (12.1) | 122  (22.4) | 574 (13.0) | 107 (9.8) | 259 (9.4) | 59 (10.8) | 425 (9.7) | 141 (12.97) | 428 (15.5) | 149 (27.3) | 718 (16.3) |
| Age, clinic adjusted  (Model 1) | 1.0 (referent) | 1.02  (0.82, 1.26) | 1.77  (1.36,  2.29) | 1.60 (1.34, 1.90) | 1.0 (referent) | 0.92 (0.73, 1.15) | 1.09 (0.79, 1.51) | 1.13 (0.9, 1.41) | 1.0 (referent) | 1.09 (0.90, 1.32) | 1.84 (1.46, 2.33) | 1.62 (1.39, 1.89) |
| Multivariate adjusted*  (Model 2) | 1.0 (referent) | 1.03 (0.83, 1.28) | 1.62  (1.25,  2.12) | 1.48 (1.25, 1.76) | 1.0 (referent) | 0.91 (0.73, 1.15) | 0.98 (0.70, 1.36) | 1.05 (0.84, 1.32) | 1.0 (referent) | 1.05 (0.87, 1.28) | 1.61 (1.27, 2.05) | 1.47 (1.25, 1.72) |
| Model 2 +  Visit 1 BMD | 1.0 (referent) | 1.03 (0.83, 1.28) | 1.62  (1.25,  2.12) | 1.48 (1.25, 1.76) | 1.0 (referent) | 0.90 (0.72, 1.13) | 0.98 (0.70, 1.37) | 1.05 (0.84, 1.32) | 1.0 (referent) | 1.05 (0.86, 1.27) | 1.61 (1.27, 2.05) | 1.48 (1.26, 1.74) |
| Model 2 +  Visit 2 BMD | 1.0 (referent) | 1.01 (0.81, 1.26) | 1.57  (1.19, 2.07) | 1.47 (1.23, 1.76) | 1.0 (referent) | 0.86 (0.68, 1.09) | 0.89 (0.63, 1.26) | 0.98 (0.77, 1.25) | 1.0 (referent) | 1.01 (0.83, 1.23) | 1.51 (1.17, 1.94) | 1.42 (1.21, 1.68) |

*Models adjusted for adjusted for Visit 1 age, clinic site, weight, physical activity, self-reported heath, presence of at least one comorbid condition, smoking status, chair stands performance, concurrent change in weight, and concurrent change in self-reported physical activity.
