# Supplementary material for: Bone Loss at the Hip and Subsequent Mortality in Older Men: The Osteoporotic Fractures in Men (MrOS) Study
Source: JBMR Plus. 2017 Jul 10;1(1):31–5. doi: 10.1002/jbm4.10006 (PMC5673261; doi:10.1002/jbm4.10006)
Supplement: Supplementary file 2 — Supporting Table S1. [file JBM4-1-31-s002.docx]

| Supplemental Table 2. Risk of cause-specific mortality by change in total hip BMD in older men. | | | | | | | | | | | | |
| --- | --- | --- | --- | --- | --- | --- | --- | --- | --- | --- | --- | --- |
|  | CVD Death | | | | Cancer Death | | | | Other Death (non cancer non CVD) | | | |
|  | BMD change category | | | per SD | BMD change category | | | per SD | BMD change category | | | per SD |
|  | Maintained | Expected | Accel. |  | Maintained | Expected | Accel. |  | Maintained | Expected | Accel. |  |
| Total n | 1150 | 2648 | 602 | 4400 (all men) | 1150 | 2648 | 602 | 4400 (all men) | 1150 | 2648 | 602 | 4400 (all men) |
| N (%) died | 112 (9.7) | 320 (12.1) | 142 (23.6) | 574 (13.0) | 94 (8.2) | 267 (10.1) | 64 (10.6) | 425 (9.7) | 140 (12.2) | 397 (15.0) | 181 (30.1) | 718 (16.3) |
| Age, clinic adjusted  (Model 1) | 1.0 (referent) | 1.08 (0.87, 1.34) | 1.98 (1.53, 2.56) | 1.77 (1.52, 2.07) | 1.0 (referent) | 1.16 (0.92, 1.47) | 1.28 (0.92, 1.78) | 1.14 (0.94, 1.39) | 1.0 (referent) | 1.08 (0.89, 1.31) | 2.08 (1.65, 2.61) | 1.75 (1.52, 2.01) |
| Multivariate adjusted*  (Model 2) | 1.0 (referent) | 1.09 (0.87, 1.36) | 1.78 (1.36, 2.33) | 1.66 (1.41, 1.96) | 1.0 (referent) | 1.15 (0.91, 1.47) | 1.09 (0.77, 1.54) | 1.05 (0.85, 1.29) | 1.0 (referent) | 1.05 (0.86, 1.28) | 1.74 (1.36, 2.21) | 1.53 (1.32, 1.78) |
| Model 2 +  Visit 1 BMD | 1.0 (referent) | 1.09 (0.87, 1.36) | 1.78 (1.36, 2.33) | 1.66 (1.41, 1.95) | 1.0 (referent) | 1.15 (0.91, 1.46) | 1.08 (0.77, 1.53) | 1.04 (0.84, 1.28) | 1.0 (referent) | 1.05 (0.86, 1.27) | 1.73 (1.36, 2.20) | 1.53 (1.32, 1.78) |
| Model 2 +  Visit 2 BMD | 1.0 (referent) | 1.07 (0.86, 1.34) | 1.71 (1.29, 2.27) | 1.64 (1.38, 1.95) | 1.0 (referent) | 1.12 (0.88, 1.42) | 1.01 (0.71, 1.44) | 0.99 (0.79, 1.23) | 1.0 (referent) | 1.02 (0.84, 1.25) | 1.64 (1.28, 2.11) | 1.49 (1.27, 1.74) |

*Models adjusted for adjusted for Visit 1 age, clinic site, weight, physical activity, self-reported heath, presence of at least one comorbid condition, smoking status, chair stands performance, concurrent change in weight, and concurrent change in self-reported physical activity.
